# Conscious Augmentation of Creative State Enhances “Real” Creativity in Open-Ended Analogical Reasoning

**DOI:** 10.1371/journal.pone.0150773

**Published:** 2016-03-09

**Authors:** Adam B. Weinberger, Hari Iyer, Adam E. Green

**Affiliations:** Department of Psychology, Georgetown University, Washington DC, United States of America; University of Georgia, UNITED STATES

## Abstract

Humans have an impressive ability to augment their creative state (i.e., to consciously try and succeed at thinking more creatively). Though this “thinking cap” phenomenon is commonly experienced, the range of its potential has not been fully explored by creativity research, which has often focused instead on creativity as a trait. A key question concerns the extent to which conscious augmentation of state creativity can improve creative reasoning. Although artistic creativity is also of great interest, it is creative reasoning that frequently leads to innovative advances in science and industry. Here, we studied state creativity in analogical reasoning, a form of relational reasoning that spans the conceptual divide between intelligence and creativity and is a core mechanism for creative innovation. Participants performed a novel Analogy Finding Task paradigm in which they sought valid analogical connections in a matrix of word-pairs. An explicit creativity cue elicited formation of substantially more creative analogical connections (measured via latent semantic analysis). Critically, the increase in creative analogy formation was not due to a generally more liberal criterion for analogy formation (that is, it appeared to reflect “real” creativity rather than divergence at the expense of appropriateness). The use of an online sample provided evidence that state creativity augmentation can be successfully elicited by remote cuing in an online environment. Analysis of an intelligence measure provided preliminary indication that the influential “threshold hypothesis,” which has been proposed to characterize the relationship between intelligence and trait creativity, may be extensible to the new domain of state creativity.

## Introduction

Creativity research has frequently followed the form of intelligence research, treating creativity as a trait, i.e., a stable attribute of which some people have more and other people have less [1–3]. A somewhat smaller though quite fruitful area of creativity research has indicated an intriguing dynamism in creativity as a state that can vary, and be deliberately augmented, within an individual across relatively short durations of time [4–15] and see [16] for relevant review). This work suggests that motivation or direction strategies consistently yield relatively large effect size improvements in creative performance across verbal and nonverbal modalities. In comparison to trait creativity research, understanding state creativity may have greater utility for ongoing efforts to bolster creative thinking and innovation [17–20]; receiving a strategy for augmenting one’s state creativity is likely to be more useful than receiving a score assessing one’s trait creativity.

Of particular importance to efforts aimed at augmenting creativity and innovation is state creativity in the context of reasoning. Creative reasoning, especially in the form of analogical reasoning, is frequently the basis of innovation in science and industry [21–26]. Analogical reasoning is a good model for creativity in reasoning [7, 22, 23, 25–30] because it can involve great divergence (highly creative analogies reveal hidden similarities between items or concepts that seem unrelated on the surface), but is also clearly constrained (in order to be valid, the pieces of an analogy must align with each other in sensible ways [31–33]. These attributes of divergence and sensible constraint make analogy a form of reasoning that meets the modern consensus definition of creativity (for review, see [34]). To our knowledge, few extant studies have investigated state creativity effects on reasoning [6, 35, 36], and these studies have targeted analogical reasoning. Green et al. [6] showed participants two word-pairs (e.g., Nose:Scent and Tongue:Taste) and asked them to indicate whether they constituted a valid four-word analogy. When explicitly instructed to think creatively, participants showed greater accuracy in correctly assessing creative analogies (e.g., Nose:Scent:: Antenna:Signal) as valid, without an accompanying increase in “false alarm” assessment of invalid analogies (e.g., Nose:Scent:: Eyelash:Mascara) as valid. Vendetti et al. [36] devised an innovative paradigm in which the creative analogy stimuli used by Green and colleagues were employed as an intervention to manipulate reasoning. Performing creative analogies successfully induced relational, rather than superficial, pairings between a highlighted item in a probe picture and an analogically related item in a second picture.

This initial evidence suggests that the dynamism of state creativity can be channeled to improve reasoning, but important questions remain. Valuable creative insights in the sciences and industry cannot often be gained by simply assessing the validity of an already-formed analogy as in the study by Green et al. [6] or by choosing a simple relational pairing rather than a superficial pairing given only two viable alternatives as in the study by Vendetti et al. [36]. Rather, creative analogical insights generally require the reasoner to seek out and find analogical connections that others may have missed among a wide array of possible concept combinations, where most of the possible combinations do not lead to valid analogies [21–26, 37, 38]. Thus, more open-ended tests of analogical reasoning are necessary to understand the relationship of state creativity augmentation to the kind of reasoning that supports innovation.

A further question concerns the relationship between state creativity and intelligence. The creativity-intelligence relationship has been a target of inquiry in the trait creativity literature. Much of this work has surrounded the “threshold hypothesis,” originally proposed by Guilford [2]. The threshold hypothesis holds that creativity correlates with IQ more strongly for people below a threshold IQ (typically around 120) than for people with an IQ that exceeds the threshold (for reviews, see [39,40]). According to this account, intelligent cognitive resources support creativity and thus greater availability of such resources generally supports greater creativity. The IQ threshold represents the point at which the cognitive resources that can support creativity are fully available or nearly so, such that additional cognitive resources have diminishing value. Above the threshold, IQ and creativity are posited to exist as largely independent variables. Though by no means a settled issue [41,42], empirical support for the threshold hypothesis comes from evidence of stronger association between creativity and IQ in low to average IQ ranges than in higher IQ ranges [39,40], including relatively recent empirical support [43,44]. However, we are not aware of any research that has tested whether the threshold hypothesis applies to state creativity. Thus, there is currently no empirical basis for characterizing how the ability to augment creative state may be related to intelligence.

An additional unexplored question concerns the effectiveness of state creativity augmentation via remote online communication. This question is timely as online communication becomes an increasingly common medium for education, work, and research [45–47]. Studies of state creativity augmentation to date have used in-person testing of participants, so it remains unknown whether creativity cues may or may not be effective when they are received remotely and without observation by an experimenter.

To contribute to the developing understanding of state creativity, and state creative reasoning in particular, we sought to address these questions. We employed a novel Analogy Finding Task devised to be sufficiently open-ended to detect individual differences in creative performance yet sufficiently constrained to enable sensitivity to inappropriate responding. The Analogy Finding Task tests participants’ ability to find analogical connections among a large matrix of word pairs while avoiding combinations of word pairs that form invalid analogies. State creativity was measured, as in prior state creativity research (e.g., [6, 7], by the effect of a cue to think creatively. We additionally obtained intelligence measures in order to preliminarily test their relationship to state creativity and, in particular, to provide an initial assessment of the threshold hypothesis for state creativity. Departing from prior state creativity research, the data were collected via online testing, enabling us to measure the effectiveness of a remote creativity cue in an online environment.

## Methods

### Participants

One hundred and fifty seven participants were recruited online via Amazon Mechanical Turk (MTurk; https://www.mturk.com/mturk/welcome) in April of 2015 and paid $2.00 for participation. Data were quality controlled to identify responses not indicative of appropriate attention to task instructions. Exclusion of a substantial number of participants is common for online data collection via Mturk and was anticipated for the present study. Thirty-seven Participants who did not complete the entirety of the survey were removed. Completion times were reviewed and 17 participants with times greater than two standard deviations above the mean (i.e., > 65 minutes) or less than one standard deviation below the mean (i.e., < 20 minutes) were considered to be potentially affected by inappropriate attention to the tasks and were removed. Note that two standard deviations below the mean would have been 0 minutes and thus not a meaningful exclusion criterion. IP Addresses were reviewed. In one case, multiple participants had the same IP address, and only the first completed survey from this IP address was included for analysis. Two participants who participated in the pilot phase of the study, described below, were also removed. A question to “prove you are human” appeared at the end of the survey. Three ostensible participants who did not accurately complete this question were removed. Lastly, quality control analogies were embedded within the Analogy Finding task itself. The task was broken into two matrices of word pairs (described below), and each matrix included 5 quality control analogies that were determined via a pilot study (described below) to be the most readily identifiable analogies in the matrices. In order to eliminate participants who were either not properly following instructions or not giving appropriate attention to task performance, seven participants who did not identify at least three quality control analogies in each matrix were removed from further analysis. The fully quality-controlled dataset included 90 participants (56.0% male; age: *M* = 35.5 ± *SD* = 9.9 years). Written informed consent from all participants (main study and pilot study) was obtained according to Institutional Review Board guidelines. Study procedures were approved by the Georgetown University Institutional Review Board.

### Pilot Study Participants

An additional group of participants was recruited through MTurk from December 2013 to March 2014 for a pilot study. This study had two main objectives. First, because our task was presented in the form of two matrices in sequence, we wanted to determine whether practice effects would occur (i.e., whether performance on the second matrix would be systematically better than performance on the first matrix). Second, we sought to confirm that a set of quality control analogies embedded in each matrix would be the most readily identifiable analogies in each matrix (i.e., correctly found more frequently than other analogies).

A total of 296 participants were recruited for the pilot study. Based on similar exclusion criteria to the ones described above, the sample was cut down to a final N of 178 (43.8% male, age 34.2 ± 12.1 years). Since we were interested in confirming that the quality control analogies would be the most readily identified items in the matrices, participants were not initially excluded for failing to identify quality control analogies.

### Materials and procedure

The Analogy Finding task consisted of two matrices of word-pairs (S1 Appendix). Each matrix contained 5 word pairs arranged in a column on the left side of the screen (stem pairs) and 20 word-pairs arranged in a row across the top of the screen (completion pairs). Participants were instructed as follows: “Your task is to make analogies by combining word pairs on the left side of the grid with word pairs along the top of the grid. Each word pair should be read as ‘[Top Word] is to [Bottom Word]’ For example, ‘Helmet is to Head.’ Check the boxes to indicate when a word pair from the top combines with a word pair on the left to make a valid analogy.” Each stem pair could be combined with 3 or 4 completion pairs to form valid analogies, such that there were a total of 17 potential valid analogies that could be found within each matrix (i.e., valid analogies formed by combining one of the stem pairs with one of the completion pairs), and 84 potential word-pair combinations that yielded invalid analogies in each matrix. There was no restriction against using the same completion pair with more than one stem pair and indeed each matrix included one completion pair that could be validly combined with two stem pairs. This was done to ensure that participants did not eliminate completion pairs from consideration, keeping the search space equivalently large throughout the task. The validity of analogies was determined by domain experts, and analogy items were largely drawn from sets of stimuli used in previous full and pilot studies in our laboratory that obtained high rates of participant accuracy. The matrices were developed into a survey template that appeared online via MTurk. Survey data were captured through Qualtrics Survey Software (Provo, Utah).

The two matrices were devised to include analogies that represented similar levels of creativity. The level of creativity represented by each analogy was quantified using a measure of semantic distance derived via latent semantic analysis (LSA [48–50]; http://lsa.colorado.edu). We used the LSA topic space of “general reading up to first year college (300 factors)” and term-to-term comparison type. This measure of semantic distance corresponds to the cosine of the angle between vectors corresponding (in our usage) to the terms of each analogy within a given semantic space, which is derived through analyses of all of the contexts in which each word tends to be present or absent in that topic space [50]. Though no unitary measure is likely to exhaustively capture the construct of creativity, semantic distance determined via LSA is a reliable and construct-valid measure of creative performance [8, 29, 51, 52], and has previously been shown to be a quantifiable measure of creative performance in analogical reasoning that correlates with subjective creativity ratings of analogies [29]. Outputs from LSA computations directly reflect semantic proximity rather than semantic distance, with values ranging from 0 to 1. Thus, for ease of interpretation, we subtracted the raw values from 1 to represent semantic distance and then multiplied by 10 so that values reflect whole numbers. The total LSA semantic distance values for all possible valid analogies was similar in the two counterbalanced matrix items, 1299 and 1328. For each stem pair of each matrix, the possible valid analogies represented a range of semantic distances. Varying the semantic distance of available analogical mappings in this way enabled us to discern individual differences in the creativity of the analogies participants identified. Quality control analogies represented the lowest semantic distance of all valid analogies in the matrices.

In full, the online testing session consisted of 7 components: (1) Demographics, (2) first Analogy Finding Task matrix, (3) second Analogy Finding Task matrix (preceded by creativity cue), (4) a multilingualism survey, (5) two timed portions of multiple-choice questions of a matching and digitized version of the redrawn Vandenberg and Kuse mental rotation tasks [53, 54], (6) a digitized version of the Multidimensional Aptitude Battery-II (MAB-II [55]) Vocabulary, and (7) a digitized version of the MAB-II Similarities. The multilingualism survey was included to obtain pilot data for a separate study and was not included in analysis for this paper. The order of presentation of the two Analogy Finding task matrix items was fully counterbalanced between participants.

### Task Instructions and Creativity Cue

Prior to viewing the first Analogy Finding Task matrix, participants were instructed as follows: “Try to make as many analogies as you can. However, only valid analogies should be listed, so don’t list analogies unless you can describe how the two word pairs are analogous.” The creativity cue was given within the instructions prior to the second Analogy Finding Task matrix. Participants were instructed, “This task is the same as the one you just finished. This time, please think creatively as you search for valid analogies. Some analogies may not be obvious right away, so be sure to look for abstract connections. However, only valid analogies should be listed, so don’t list analogies unless you can describe how the two word pairs are analogous.”

### Intelligence measures

Following the Analogy Finding Task, participants completed a brief battery of intelligence measures. The Similarities and Vocabularies subscales of the Multidimensional Aptitude Battery-II (MAB-II; Jackson [55]) were used to obtain a preliminary a measure of verbal ability, including an estimated total Verbal IQ. The MAB-II subscales are closely modeled after those of the Wechsler Adult Intelligence Scales-Revised (WAIS-R; [56]). However, the MAB was designed specifically to enable automated and group administration [57]. The MAB has been validated as a suitable alternative to the WAIS-R for measurement of verbal or general abilities [58]. Performance on the Similarities and Vocabulary subtests are correlated with WAIS-R total Verbal IQ scores at .60 and .74 respectively [58]. Per the MAB-II manual, we calculated a prorated estimate of total verbal IQ from the subscales we administered [55].

A version of the redrawn Vandenberg and Kuse Mental Rotation Test (MRT; [53, 54]) was used to assess spatial ability. On each MRT trial, participants identified which, if any, of a set of complex figures was a rotated version of a probe figure [54]. Participants completed two timed sections of MRT trials. MRT performance is highly related to other overall and perceptual IQ measures [59, 60].

### Pilot Study Procedure

Participants in the pilot study completed the two Analogy Finding Task matrices used in the main study, with the order of the matrices fully counterbalanced between subjects. Participants were randomly assigned to one of two conditions: they were either cued to be creative for both matrices (Cued condition), or were not cued to be creative in either matrix (Uncued condition).

### Outcome Measures

Creativity was assessed via the following outcomes measures: (1) the total semantic distance of valid analogies identified, (2) the number of valid analogies identified, which is related but not informationally identical to the total semantic distance, and (3) the number of invalid analogies identified. The change in these measures from the first/uncued matrix to the second/cued matrix was taken to measure the effect of the creativity cue in the main study. The total, rather than the average, semantic distance was used because averages were deemed likely to provide misleading representations of the ability to formulate creative analogies. For example, it was possible to identify very few analogies (as few as one) and yet to have a high average semantic distance.

## Results

### Pilot Study

The pilot study revealed that a set of analogies intended to be used as quality control analogies in the main study were, as hoped, the most frequently identified analogies. On average, participants (N = 178) identified 8.17 out of a possible 10 quality control analogies compared with 5.73 out of a possible 24 cross-domain analogies. This difference in frequency of identification was significant (*X*^2^ = 99.10, *p* < 0.001, *r* = 0.75), and the least frequently identified quality control item was identified more often than the most frequently identified non-quality control analogy (*X*^2^ = 25.17, *p* < 0.001, *r* = 0.38).

We next investigated practice effects in the pilot study. Consistent with the main study (see below), 29 participants who failed to identify at least three quality control analogies in each matrix were removed. The reduced sample (N = 149; 40.3% male; age 34.7 ± 12.4 years), showed no practice effect of performing a second Analogy Finding Task matrix after performing a first matrix. That is, there was no significant change on any outcome measure from the first matrix to the second in either condition (i.e., when both matrices were cued, or when both matrices were not cued; all *p* > .153; Table 1).

**Table 1:** Pilot study data indicating no practice effects. Means with standard deviations in parenthesis for each outcome measure, and p-values for comparisons between matrices. No significant differences were found for any of the outcome measures between the counterbalanced first and second matrices when both matrices were performed with the creativity cue (Cued condition) or when both matrices were performed without the creativity cue (Uncued condition), indicating no practice effects.

	Total Semantic Distance	Number of Valid analogies	Number of Invalid Analogies
**Cued condition N = 69**			
Matrix 1	562.93 (317.03)	8.32 (3.69)	3.35 (4.00)
Matrix 2	565.04 (301.01)	8.36 (3.56)	3.93 (5.11)
p-value	0.934	0.883	0.153
**Uncued condition N = 80**			
Matrix 1	483.70 (287.50)	7.46 (3.34)	2.59 (3.70)
Matrix 2	459.06 (284.34)	7.13 (3.29)	2.68 (3.78)
p-value	0.348	0.254	0.781

Finally, the pilot study data provide a between-subjects confirmation of the within-subjects findings of the main study concerning the effect of the creativity cue. Valid analogies identified by Pilot participants in the Cued condition (when both matrices were cued; N = 69) were more semantically distant than those identified by participants in the Uncued condition (when neither matrix was cued; N = 80; both p ≤ .010).

## Main Study

### Effect of the creativity cue

**Fig 1:**
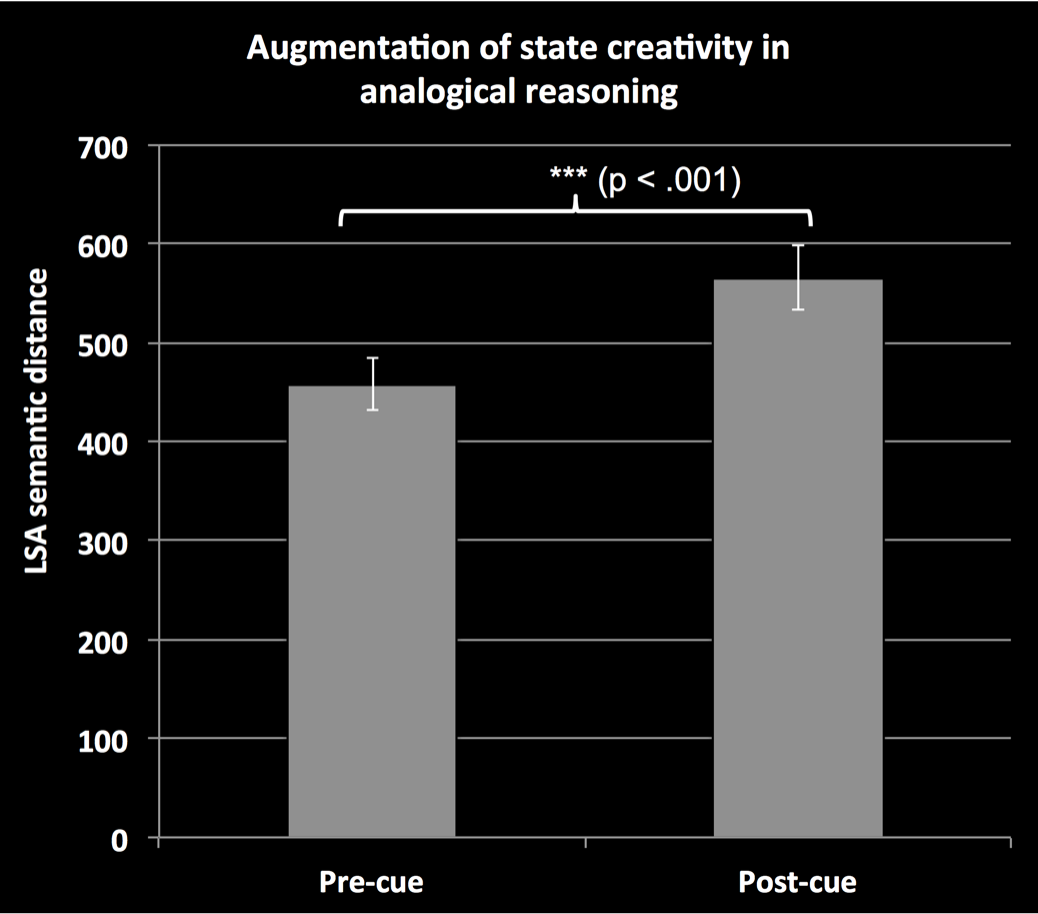
Effect of an explicit creativity cue on semantic distance in analogical reasoning. Total semantic distance of analogies formed in the Analogy Finding Task matrices before and after the creativity cue. The cue elicited the formation of substantially more creative (semantically distant) analogies. Error bars represent one standard error of the mean.

Consistent with our primary hypothesis, participants showed notable improvement on creativity measures following the creativity cue. Participants identified analogies that were more creative (greater semantic distance) in the second/post-cue matrix (*M* = 565.33 ± *SD* = 305.96) than in the first/pre-cue matrix (*M* = 457.69 ± *SD* = 247.09), *t*(89) = 5.48, *p* < .001, *d* = 0.39 (Fig 1). Participants identified a greater number of valid analogies on the second/cued matrix (*M* = 8.38 ± *SD* = 3.52) than on the first/uncued matrix (*M* = 7.12 ± *SD* = 2.91), *t*(89) = 5.90, *p* < .001, *d* = 0.39. Critically, this increase in finding valid analogies was not accompanied by a greater number of “false alarm” invalid analogies identified in the second/cued matrix (*M* = 2.23 ± *SD* = 3.22) than in the first/uncued matrix (*M* = 1.98 ± *SD* = 3.42), *t*(89) = .97, *p* = .334. A repeated measures ANCOVA model taking number of false alarm analogies as a covariate demonstrated that the effect of the creativity cue on semantic distance was independent of false alarms, *F*(1, 88) = 31.08, *p* < .001, *η*^2^ = .25. There was no main effect of false alarms in this model (*p* = .752), and no interaction of cue by false alarms (*p* = .266). Additionally, a linear regression model confirmed that pre-cue vs. post-cue change in the number of false alarm analogies was not predictive of the change in total semantic distance, *β* = .10, *t*(88) = .965, *p* = .337.

#### Intelligence measures

Participants showed an average estimated Verbal IQ of 114.19 ± *SD* = 13.23, and an average MRT score (as determined by percent correct) of 39.54% ± *SD* = 22.89%. Verbal IQ was strongly correlated with the change in semantic distance from the pre-cue to the post-cue matrix (effect of the cue; *r* = .38; *p* < .001). MRT score also showed a correlation with change in semantic distance that approached significance (effect of the cue; *r* = .20; *p* = .053). A model in which change in semantic distance (effect of cue) was regressed on both Verbal IQ and MRT indicated that Verbal IQ remained significantly predictive, *β* = .35, *t*(87) = 3.27, *p* = .002, whereas MRT was not significantly predictive in this model *β* = .08, *t*(87) = .77, *p* = .441. First-order correlations of the two intelligence measures with all creativity outcome measures are reported in S1 Table.

#### Assessing an IQ threshold effect

To test whether an IQ threshold effect could be observed for augmentation of state creativity in our data (i.e., change in creativity after the cue), the dataset was split into groups of participants with higher and lower Verbal IQ. We used Verbal IQ ≥ 120 as the threshold for inclusion in the higher IQ group based on prior research [2, 3, 42, 44]. The higher IQ group included 35 participants with average Verbal IQ = 126.71 ± *SD* = 4.24. The lower IQ group included 55 participants with average Verbal IQ = 106.22 ± *SD* = 10.72. A 2(Cue: Pre, Post) X 2(IQ group: Higher, Lower) ANOVA indicated an expected main effect of the creativity cue on semantic distance (*F*(1, 88) = 39.43, *p* < .001, *η*^2^ = .31), a main effect of IQ group (*F*(1, 88) = 13.20, *p* < .001, *η*^2^ = .13), and, most relevant to the threshold effect, an interaction of Cue by IQ group (*F*(1, 88) = 9.51, *p* = .003, *η*^2^ = .10) indicating that the creativity cue yielded greater increases in semantic distance in the higher IQ group.

In the lower IQ group, change in semantic distance (effect of cue) was significantly correlated with Verbal IQ (*r* = .36, *p* = .007). By contrast, the higher IQ group showed no correlation between change in semantic distance and Verbal IQ (*r* = .01, *p* > .949). Fisher’s r-z transformations suggested a difference between IQ groups for these correlation coefficients that approached significance (*p* = .053 one-tailed).

## Discussion

We found that an explicit creativity cue successfully elicited an augmentation of state creative performance in a novel open-ended analogical reasoning task. In a matrix of potential word-pair combinations, participants formed more creative analogies after receiving a cue to think creatively than before receiving the cue (creativity was measured by LSA-derived semantic distance), and formed a greater number of valid analogies. Critically, the increase in formation of creative analogies was not due to an increase in false alarm formation of invalid analogies, and invalid analogies were not more numerous after the creativity cue. This indicates that the augmentation of state creativity we elicited does not reflect an augmentation of divergence at the expense of reasonable constraint (i.e., augmented state creativity in analogical reasoning appears to be “real” creativity; [34].

The present findings extend previous work demonstrating that creativity cueing can elicit conscious augmentation of state creativity [4–13]. A notable element of the present study was the open-ended paradigm we used to instantiate creative reasoning. Each Analogy Finding Task matrix contained 100 possible word-pair combinations, 83 of which did not result in valid analogies. The requirement to selectively search out first-order relations (represented by individual word-pairs) and combine them to form valid second-order relations (i.e., analogies) differed substantially from the one extant study to our knowledge that has investigated conscious (i.e., explicitly cued) augmentation of state creativity in reasoning [6]. That study employed an evaluation paradigm in which participants simply provided yes or no responses to indicate whether fully-formed four-term stimuli represented valid analogies. What distinguishes creative analogical thinkers in the real world is the ability to find non-obvious analogical connections between available information; that is, to selectively search out first-order relations in an information-rich environment to form valuable second-order relations that can yield innovation [21–26, 37, 38]. Thus, the present paradigm represents a meaningful step toward greater ecological validity in the study of state creative reasoning.

Another notable aspect of the present study was the use of a remote creativity cue via online participant interface. We are not aware of any prior study of state creativity that has used a remote paradigm. Evidence that a remote cue can effectively elicit augmented state creative performance is encouraging, and suggests that our creativity cueing approach may be adaptable to improve creative performance in a wide array of online interactions for teaching and ideation in science, arts, and industry [45–47]. Online testing via Amazon MTurk also enabled a measurement of cued state creativity augmentation in a participant pool that is generally much more socio-economically and ethnically diverse than the typical cohorts of university students and community members that populate many studies in the behavioral and cognitive sciences [61]. Nonetheless, data obtained via Mturk testing have been shown to be similarly reliable to data obtained via more traditional face-to-face methods [62].

There are, however, drawbacks and potential interpretive confounds associated with online testing. We implemented several rounds of quality control to attempt to address potential confounds (e.g., ensuring that respondents were human, that they finished the survey, that the time they spent working on the tasks indicated sustained engagement and meaningful responding, and that identification of quality control analogy items indicated appropriate attention to accuracy). In addition, the Analogy Finding Task was not easily susceptible to inappropriate use of outside materials; the correct answers could not be found in any external resource. Other confounds are more difficult to address, including potential interactions of varying testing environments with our creativity cue manipulation.

Analysis of the relationship between a measure of Verbal IQ and augmentation of state creative performance indicated preliminary support for a threshold effect. We found an interaction of cue by IQ group on the semantic distance of analogies, and the correlation between IQ and cued augmentation of creative performance was substantially higher in the lower IQ group. This research is a first empirical exploration of the threshold hypothesis in the context of state creativity. Taken together, the findings provide a preliminary indication that the threshold hypothesis may extend into this novel domain.

Some prior research has questioned the validity of the threshold hypothesis. Runco and Albert [41] concluded that the theory may be a “psychometric artifact” after discovering nonsignificant correlations between creativity and achievement in a low achieving group of intermediate school children but significant correlations in the high achievement group and Preckel et al. [43] reported comparable correlations between creativity and intelligence across IQ ranges. A clear limitation on the conclusions we can draw concerning the threshold hypothesis in the present study comes from the use of an abbreviated measure to estimate verbal IQ. While the MAB is designed for remote implementation, and is a reasonably good predictor of full-scale IQ on a more widely-used scale (WAIS-R), our measure should only be taken as approximate indicator, and was only intended to enable an initial sounding for a threshold effect in state creativity. One potential concern with our remote IQ measure is that, unlike the Analogy Finding Task, it was possible for participants to use external resources to find definitions for the words used in the MAB Vocabulary and Similarities subscales. It should be noted that the use of a somewhat nonstandard measure of intelligence is not uncommon in the extant literature on the threshold hypothesis. Previous research has used a wide range of instruments to measure intelligence. A meta-analysis of the relationship between creativity and IQ [40] indicated that a wide variety of tests are used to assess IQ in the context of creativity, including measures limited to verbal-domain intelligence. Thus, our measure of Verbal IQ is not out of place in a literature that could generally benefit from greater uniformity to aid in clearer determinations concerning the validity of the threshold hypothesis.

## Conclusions

Augmentation of state creativity to improve creative reasoning is a promising research direction with potential to inform strategies for innovation in multiple contexts. Here, we extended prior evidence of successful conscious augmentation of creative state in response to an explicit creativity cue. The data provide new evidence that conscious state creativity augmentation can be extended to formation of analogies in an open-ended workspace, and that remote cueing of state creativity is effective in an online environment. A key element of the present findings was that increases in creative analogy finding appear to reflect “real” creativity rather than simply augmented divergence at the expense of sensible constraint. The present data also provide preliminary evidence that the threshold hypothesis, which has been influential in understanding their relationship between intelligence and trait creativity, may be extensible to the domain of state creativity. This work sets the stage for further work to test the boundary conditions of the efficacy of state creativity augmentation in reasoning, including exploration of this phenomenon in increasingly ecologically valid paradigms.

## Supporting Information

S1 Appendix Analogy Finding Task matrix items. (DOCX)

S1 Table Correlations between intelligence measures and creativity cue-related changes in creativity outcome measures. (DOCX)
