# Supplementary figures and images for: Conscious Augmentation of Creative State Enhances “Real” Creativity in Open-Ended Analogical Reasoning
Source: PLoS One. 2016 Mar 9;11(3):e0150773. doi: 10.1371/journal.pone.0150773 (PMC4784911; doi:10.1371/journal.pone.0150773)

**S1 Appendix.**

**Analogy Finding Task matrices.**


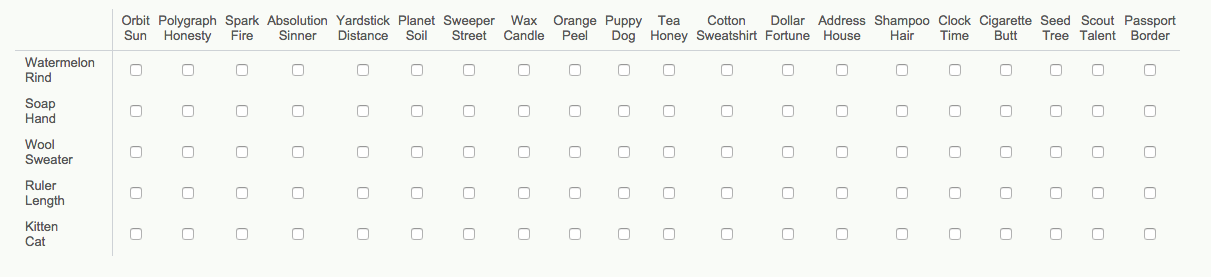


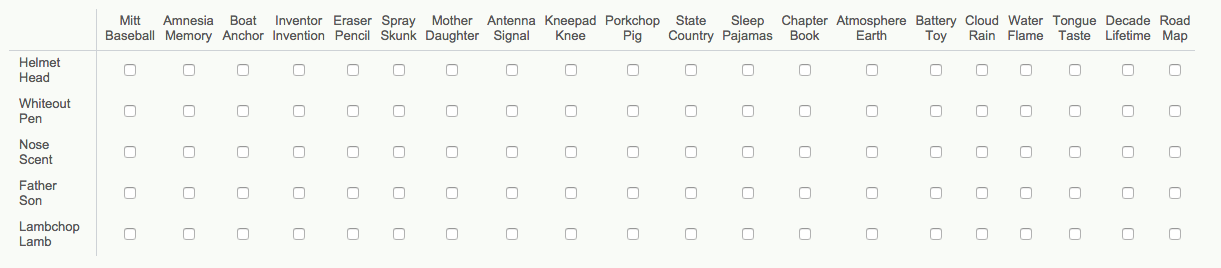

Supplement: S1 Appendix — (DOCX) [file pone.0150773.s001.docx]
