# Supplementary material for: Conscious Augmentation of Creative State Enhances “Real” Creativity in Open-Ended Analogical Reasoning
Source: PLoS One. 2016 Mar 9;11(3):e0150773. doi: 10.1371/journal.pone.0150773 (PMC4784911; doi:10.1371/journal.pone.0150773)
Supplement: S1 Table — (DOCX) [file pone.0150773.s002.docx]

| **S1 Table. Correlations between intelligence measures and creativity cue-related changes in creativity outcome measures.** | | | | | | |
| --- | --- | --- | --- | --- | --- | --- |
|  | | Change in Semantic Distance | Change in Correct Analogies Found | Change in Incorrect Analogies Identified | MRT Score | Verbal IQ |
| Change in Semantic Distance | Pearson Correlation | 1 |  |  |  |  |
|  | Sig. (2-tailed) |  |  |  |  |  |
|  | N | 90 |  |  |  |  |
| Change in Correct Analogies Found | Pearson Correlation | .964^**^ | 1 |  |  |  |
|  | Sig. (2-tailed) | .000 |  |  |  |  |
|  | N | 90 | 90 |  |  |  |
| Change in Incorrect Analogies Identified | Pearson Correlation | .102 | .109 | 1 |  |  |
|  | Sig. (2-tailed) | .337 | .307 |  |  |  |
|  | N | 90 | 90 | 90 |  |  |
| MRT Score | Pearson Correlation | .204 | .194 | .163 | 1 |  |
|  | Sig. (2-tailed) | .053 | .067 | .125 |  |  |
|  | N | 90 | 90 | 90 | 90 |  |
| Verbal IQ | Pearson Correlation | .375^**^ | .358^**^ | .287^**^ | .354^**^ | 1 |
|  | Sig. (2-tailed) | .000 | .001 | .006 | .001 |  |
|  | N | 90 | 90 | 90 | 90 | 90 |
| **. Correlation is significant at the 0.01 level (2-tailed). | | | | | | |
